# A Family Physician’s Experience in the Removal of Migrating Subdermal Contraceptive Implants

**DOI:** 10.7759/cureus.68264

**Published:** 2024-08-31

**Authors:** Razaz Wali, Maryam S BinBakr

**Affiliations:** 1 Primary Healthcare, Ministry of National Guard Health Affairs, Jeddah, SAU; 2 Family Medicine, King Abdullah International Medical Research Center, Jeddah, SAU; 3 Family Medicine, King Saud bin Abdulaziz University for Health Sciences, Jeddah, SAU

**Keywords:** subdermal implant, removal, migrating, family physicians, contraceptive

## Abstract

Subdermal contraceptive implants are among the most effective and reversible contraceptive methods available today. Implanon®️, approved by the US FDA, is widely used for long-term family planning due to its extended contraceptive effects. Although generally safe, these implants can occasionally lead to rare but serious complications, such as migration into deeper axillary structures. In this case report, we describe the experience of a family physician with a 30-year-old Saudi woman whose Implanon implant migrated approximately 11 cm from the original insertion site over three years. The patient presented for removal of the implant, which had reached the end of its contraceptive duration and was to be removed in preparation for future conception. Initial attempts by the family physician to localize and remove the implant were unsuccessful due to its continued migration toward the axilla. Consequently, the case was referred to the obstetrics and gynecology clinic and subsequently to the orthopedic department. The foreign body was successfully removed using an intraoperative, fluoroscopy-guided procedure, with no complications observed post-surgery. This case underscores the importance of physician awareness and a multidisciplinary approach to managing such complications. It also highlights that implant migration is not necessarily related to the physician’s skill or patient characteristics, underscoring the unpredictable nature of this complication. This report aims to provide insights and recommendations for handling similar cases, advocating for early intervention, and the use of imaging techniques when managing non-palpable implants.

## Introduction

Subepidermal contraceptive implants are a reliable and essential choice for family planning, valued for their extended contraceptive effects lasting up to three years. Implanon®️, approved by the US FDA in 2006 and manufactured by Merck & Co., Inc. (formerly Organon USA, Inc., Kenilworth, NJ, USA), functions by releasing progesterone to inhibit ovulation and alter vaginal mucus, thus preventing sperm movement and pregnancy [1].

Implants are inserted about 8-10 cm from the medial epicondyle of the humerus in the non-dominant arm through a small incision. Although insertion is straightforward, it requires proper training to ensure correct placement and depth. Minor implant displacements are common and generally easy to manage, but significant migrations can complicate removal, sometimes necessitating surgical intervention [2].

Family physicians, as first-line healthcare providers, frequently encounter women during pregnancy and the postpartum period - an ideal time to discuss and initiate contraception, including the use of subdermal implants. It is crucial for these physicians to be trained in both the insertion and removal of subdermal implants [3].

While multiple case reports have documented subdermal implants migrating to various body parts, such as the pulmonary vasculature, lungs, and soft tissue [4,5], few have addressed the physician’s experience in managing such complications. This case report highlights a rare instance involving a family physician’s expertise in dealing with a migrating subdermal contraceptive implant during removal. It aims to share this experience with global colleagues, underscoring the importance of physician awareness and meticulous management in handling such issues.

## Case presentation

Case description

A 30-year-old female patient, para 2 (P2+0), with a history of uncomplicated pregnancies and vaginal deliveries, presented for the removal of an Implanon® (subdermal progesterone implant). She was a housewife with no significant medical or surgical history, a non-smoker, and not physically active. The patient was vitally stable, with physical measurements including a height of 156 cm, a weight of 42 kg, and a BMI of 17.26, indicating slightly underweight.

Case history

The patient, a 30-year-old Saudi female, presented to the family planning clinic for the removal of the Implanon® implant, which had been inserted three years prior (December 2020). During this period, she had not attended the clinic for consultations or checkups. The removal was sought due to her desire to conceive again, as the implant had reached the end of its three-year duration as specified in the Implanon leaflet. The patient had a nonsignificant clinical history and physical examination, aside from the noted implant migration.

**Figure 1:**
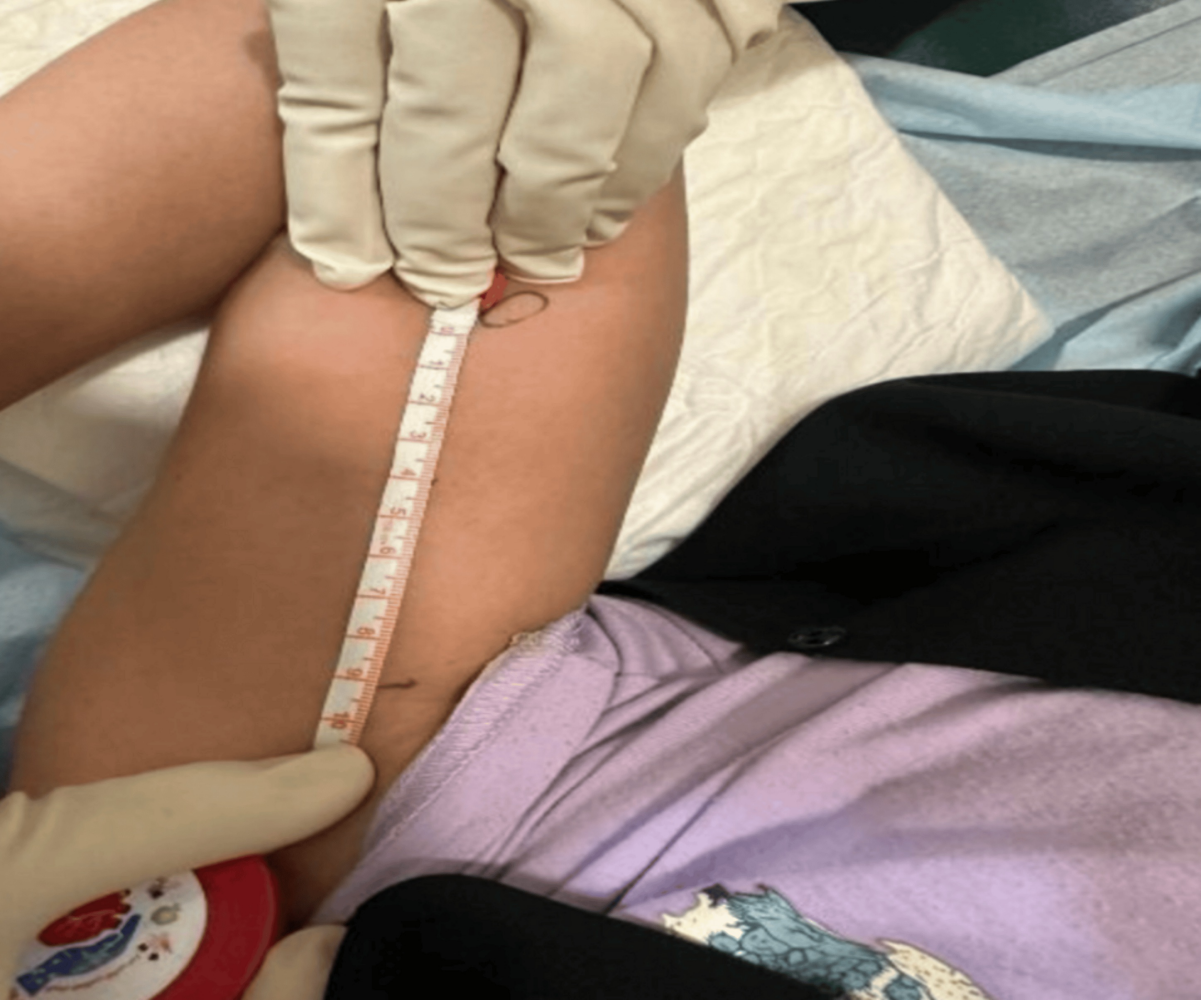
Extent of subdermal implant migration from the original insertion site Continuous migration of the subdermal implant during multiple removal attempts.

The standard procedure for subdermal implant removal involves cleaning the area, injecting local anesthesia, and marking the incision site. During the initial examination to locate the incision site, a partially palpable subdermal implant was found, with the distal edge positioned 11 cm from the original insertion scar (Figure 1). As the physician attempted to inject local anesthesia at the new location and make an incision, the implant continued to migrate proximally. Despite attempts to cut at the new site, the implant kept moving toward the axilla with each attempt. Multiple efforts to push the proximal end for easier access were unsuccessful, and eventually, the proximal end could no longer be palpated. Consequently, the procedure was aborted. This was the first incident out of 680 subdermal implant removals at the clinic over the past five years where an implant had migrated more than 11 cm from the initial insertion site (Figure 1).

Diagnostic assessment

The family physician decided to refer the case to the OB/GYN clinic for potential surgical removal.

**Figure 2:**
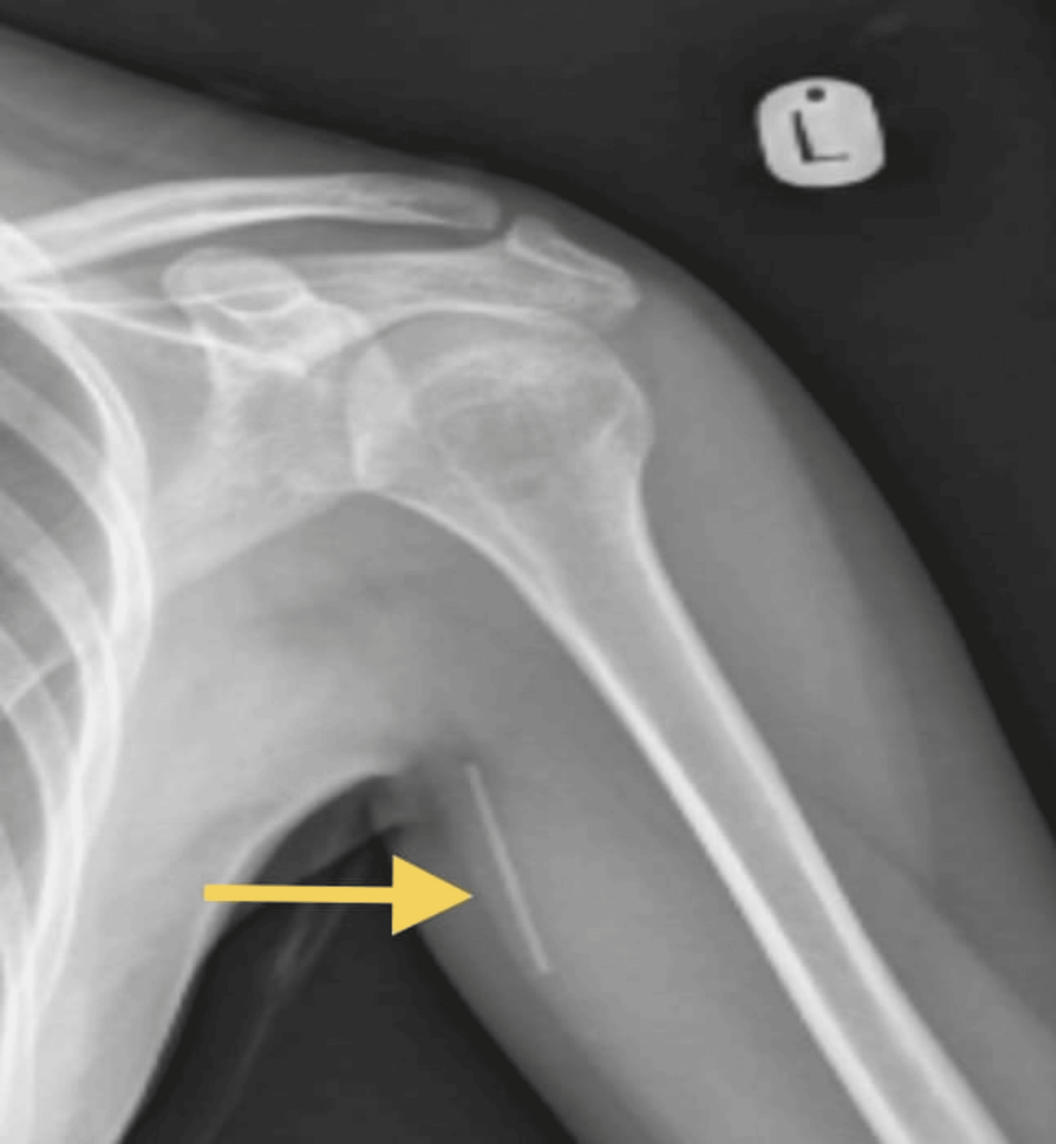
X-ray of the upper extremity showing a linear radiopaque structure in the left upper arm soft tissue, consistent with the characteristics of an implant

In the OB/GYN clinic, the gynecologist attempted to remove the implant, which was located about 5 cm proximal and deeper in the axilla with only the tip palpable. Subsequent removal attempts by the gynecologist were unsuccessful. An X-ray was ordered, revealing the implant’s location in the axilla (Figure 2). This prompted a referral to the orthopedic department for further management, in accordance with the hospital’s policy. One week later, the patient was seen in the orthopedic clinic, where an appointment for the surgical removal of the implant was scheduled.

**Figure 3:**
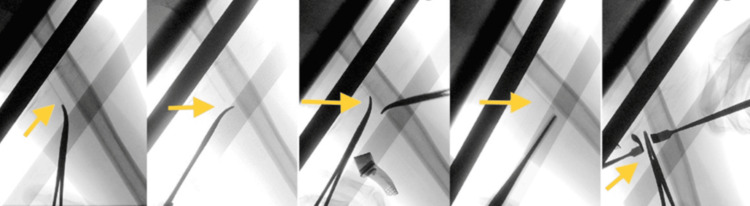
Implant removal procedure using fluoroscopy The image shows the orthopedic surgeon using a fluoroscope, a specialized X-ray machine, to enhance visualization during the removal process.

The patient was scheduled for a one-day surgery in the orthopedic clinic to remove the implant. On the day of the operation, she was admitted, and an intraoperative fluoroscopy-guided procedure was successfully performed to remove the implant (Figure 3).

**Figure 4:**
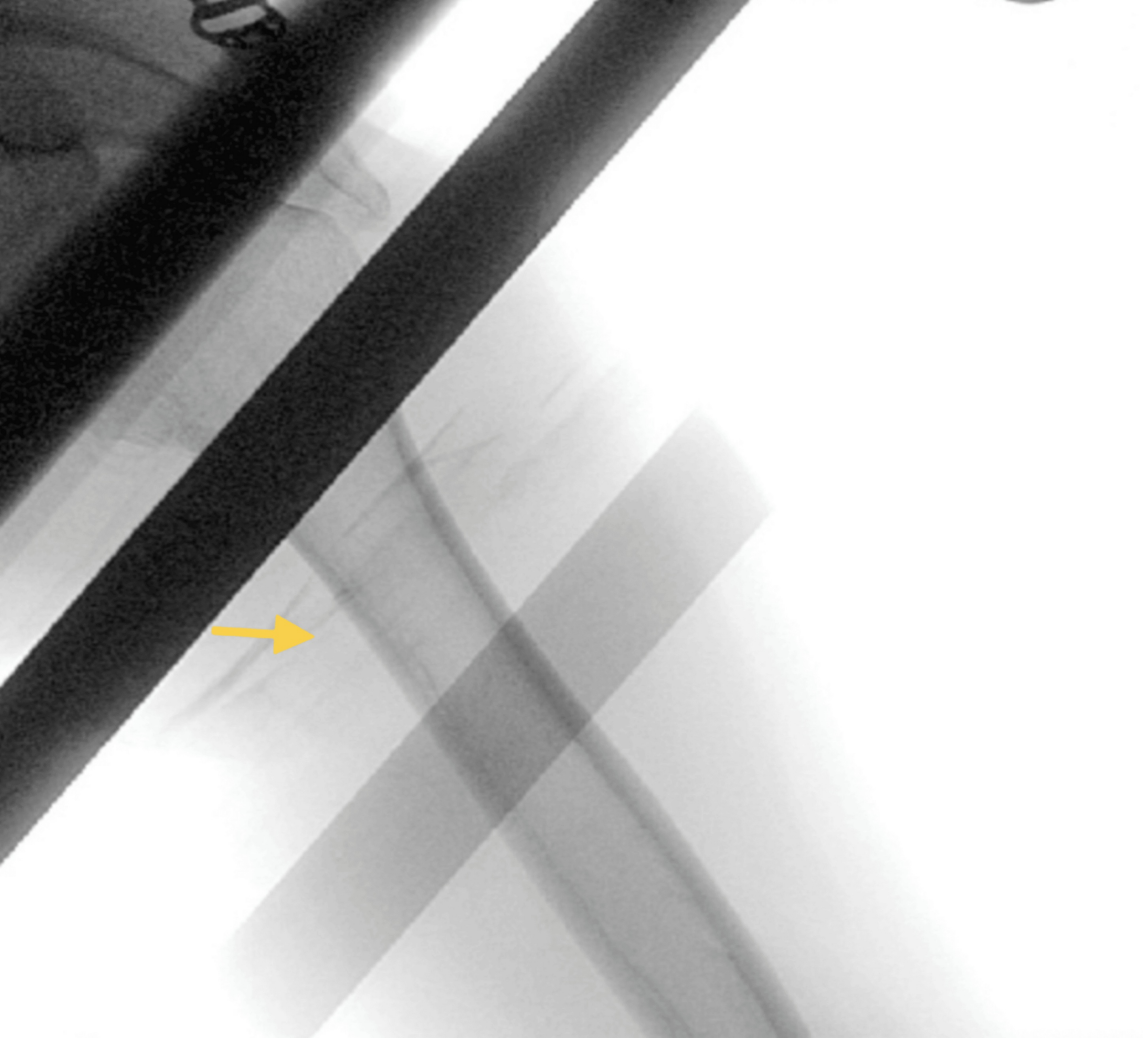
Fluoroscopy image post-procedure confirming complete implant removal The image shows the area after the implant has been removed by the orthopedic surgeon.

A follow-up X-ray confirmed the complete removal of the implant (Figure 4). The surgical site was closed without complications, and the patient was discharged with analgesics and Augmentin.

Follow-up and outcomes 

Two weeks later, during follow-up, the patient was doing well, and her wound had healed completely.

## Discussion

Subdermal contraceptive implants are highly effective for long-term contraception but can have hormonal side effects such as acne, mood changes, and headaches. More critically, they can cause rare but serious complications, such as migration into axillary structures, including muscles, fascia, and blood vessels [1].

Risk factors for Implanon migration include improper placement technique, vigorous physical activity post-placement, and deep insertion, which may lead to migration into the axilla. The axilla is an anatomically complex region with vital structures such as the brachial plexus, which innervates the upper limbs, and axillary blood vessels that connect directly to the lungs [6]. Thus, managing foreign bodies in this area can be surgically challenging.

Literature on these complications is limited, but some case reports provide insight. For example, Park et al. (2017) [7] documented an implant migrating near the brachial plexus, causing neurological symptoms that were successfully addressed through C-arm guided removal. Belyea et al. (2017) [8] described an implant located in the brachial neurovascular sheath, which, if untreated, could lead to severe neurological consequences such as chronic ulnar neuropathy and associated deformities. Alotaibi and Al-Otaibi (2024) [9] reported a case from Saudi Arabia highlighting the axillary migration of an implant, underscoring the geographical and clinical relevance of this issue.

Additional case reports have noted migration into the pulmonary vasculature [4,5]. Studies suggest that no definitive personal factors at the time of insertion can predict the likelihood of implant migration [10]. A 2022 study proposed that low BMI and weight after insertion could be a risk factor for migration, but this was not observed in this case [10]. These findings emphasize that complications are not necessarily related to the physician’s skill or experience, as even experienced professionals have faced similar issues. In this case, all known risk factors were absent, suggesting the migration was likely idiopathic.

## Conclusions

The literature reveals limited documentation on physician experiences with migrating implants, highlighting the need for improved understanding and management strategies. In this case, factors contributing to migration, such as improper placement technique, significant weight changes, and variations in patient activity levels, require further investigation to refine implantation protocols and mitigate complications.

This case report underscores the unpredictable nature of implant migration during removal and advocates for continued research to identify risk factors and enhance clinical practices. Enhanced awareness and a multidisciplinary approach are essential for achieving successful outcomes in managing the complexities of contraceptive implant therapy.
